# Editorial: New strategies and technologies enabling point of care diagnosis of neglected or tropical diseases

**DOI:** 10.3389/fcimb.2022.1089088

**Published:** 2022-12-22

**Authors:** Alexandre Dias Tavares Costa, Jacqueline Ferreira Leite Santos

**Affiliations:** ^1^ Laboratório de Ciências e Tecnologias Aplicadas à Saúde (LaCTAS), Instituto Carlos Chagas (ICC), Fundação Oswaldo Cruz (Fiocruz), Curitiba, Brazil; ^2^ Laboratório de Materiais Aplicados e Interfaces (LAMAI), Instituto de Química (IQ), Universidade Federal do Rio Grande do Sul (UFRGS), Porto Alegre, Brazil

**Keywords:** neglected and tropical diseases, rapid diagnostic tests (RDT), point of care, cerebral malaria, mitochondria-derived small RNAs, tuberculosis, alphafetoprotein (AFP), leishmania infantum

## Introduction

Diagnostics are a central tool in healthcare. However, the long time to get a reliable diagnostic may delay the treatment leading to complications. Point-of-care diagnostics (POC-Dx) became known as an interesting strategy to overcome the limitation of the conventional tests. POC-Dx is a comprehensive approach to diagnostics which should allow laboratory-exclusive techniques to be used virtually anywhere where care is needed despite the absence of adequate healthcare infrastructure or specialized personnel, therefore, delivering decentralized, fast, sensitive, low-cost, and remote diagnostics.

The development of POC tests is expanding enormously, accelerating during the COVID-19 pandemic (Bruijns et al., 2022). The development of these devices has been beneficiated by the integration and advances in nanotechnology, materials science, biotechnology, microfluidics, and different transduction methods, such as electrochemical or optical. Indeed, although several reliable devices are in the market for COVID-19 diagnosis, scientists still need to make efforts to attend to the current demands concerning neglected or tropical diseases (NTD) (Valera et al., 2021).

Currently, DNA/RNA-based POC technologies such as LAMP and qPCR have already been identified as promising tools in the diagnosis and management of NTDs (García-Bernalt Diego et al., 2021; Yalley et al., 2022). However, they have limited real-world use because sample preparation, which plays a vital role in POC-Dx, is still a significant obstacle for obtaining nucleic acids with enough quality for accurate and specific results (Ali et al., 2017). Although efforts have been made (Ali et al., 2020; Hernandez et al., 2022), a simple and effective sample preparation method or portable instrument is still a major challenge for the widespread use of POC-Dx, especially considering the economic conditions of the countries that would most need such tests (Land et al., 2019). However, new materials and reagents, as well as new instruments and technologies, are being developed to overcome such hurdles (Ferreira et al., 2009; Escobedo et al., 2013; Perez-Guaita et al., 2018; Rampazzo et al., 2019; Cereda et al., 2022).

This Research Topic features articles presenting the development of new materials, systems, and technologies in the pursuit of an efficient POC-Dx. These trends are highlighted below.

New reagents, such as new antigenic proteins that allow improved detection of *Leishmania infantum* DNA in canine samples using an immunological-based test (Ejazi et al.). They used the simplest POC test, the the rapid diagnostic tests (RDTs), to diagnose visceral leishmaniasis in dogs, in a multicentric study in different countries. Results showed particularly good sensitivity and specificity, strongly supporting a tool that would need little further investments to become a product helping public health agents.

New strategies for signal generation and amplification, such as modifying the chemical architecture of electrochemical biosensors (Chanarsa et al.). These authors used exfoliated two-dimensional (2D) molybdenum diselenide (MoSe_2_) and 2D tungsten diselenide (WSe_2_) to modify screen-printed carbon-based electrodes. This heterojunction provides a large surface area and good adsorption capacity for the immunoassay, allowing the development of a simple, reliable, and label-free immunosensor on a smart phone, by connecting to a small potentiostat. This proof-of-concept electrochemical sensor can be further developed to detect other biological targets, and one could expect the same increase in sensitivity.

New algorithms, aiming the development of a reliable tool for the identification of malaria severity and cerebral malaria. Omar et al. used machine learning algorithms to classify blood gene signatures to make this distinction. Although the study had some limitations, the authors bring an important contribution to the field through new molecular studies and biomarkers discoveries. They innovate by coupling Regularized Random Forest model (RRF) and k-top scoring pairs (K-TSP) algorithms with multi-cohort analysis to identify gene signatures capable of distinguishing cerebral and severe malaria, contributing for blood diagnostic of this tropical disease in endemic countries.

New technologies, such as a web-based app that empower clinicians with access to the internet to reliably confirm the patient’s tuberculosis diagnosis. The concern with reliable devices for the diagnosis of pulmonary tuberculosis led Ling et al., to use machine learning to diagnose tuberculosis by identifying mitochondria derived small RNAs (mtRNAs) on clinical samples. They demonstrated the development of a new model to predict tuberculosis evolution based on peripheral blood mtRNAs and a ratio-based method to improve the sensitivity and specificity of classifications between normal and tuberculosis patients. Finally, they have implemented a web-based R/Shiny app for pulmonary tuberculosis screening allowing independent investigations and faster results.

In a nutshell, NTD diagnosis needs all help available, whether through freshly new or decades-old technologies. The present Research Topic is a clear example that there is room for all ideas, strategies, and technologies in the challenging task of enabling POC-Dx for neglected or tropical diseases.

## Author contributions

AC and JS equally drafted and critically revised the manuscript for all intellectual content, and are accountable for all aspects of the work. All authors contributed to the article and approved the submitted version.
